# Nitrogen enrichment and vascular plant richness loss reduce bryophyte richness

**DOI:** 10.1038/s41598-025-88425-2

**Published:** 2025-02-03

**Authors:** Maeve Lin, Ariel Bergamini, Noémie A. Pichon, Eric Allan, Steffen Boch

**Affiliations:** 1 Swiss Federal Institute for Forest, Snow and Landscape Research WSL, Birmensdorf, Switzerland; 2 Institute of Plant Sciences, University of Bern, Bern, Switzerland; 3 Centre for Development and Environment, University of Bern, Bern, Switzerland; 4 Oeschger Centre for Climate Change Research, University of Bern, Bern, Switzerland

**Keywords:** Biodiversity experiment, Bryophyte diversity, Functional traits, Moss, Nitrogen enrichment, Species richness, Grassland ecology, Biodiversity, Conservation biology, Community ecology, Plant ecology

## Abstract

Grasslands’ high diversity is threatened by land-use changes, such as nitrogen fertilization, leading to productive but low-richness, fast-growing plant communities. Bryophytes are a key component of grassland diversity and react strongly to land use. However, it is unclear whether land-use effects are direct or mediated by changes in vascular plants. Increases in vascular plant cover are likely to decrease bryophyte abundance through light competition. Whether changes in vascular plant composition and richness also play a role remains unclear. We sampled bryophytes in a factorial grassland experiment manipulating nitrogen fertilization, fungicide, species richness, and functional composition of vascular plants crossed with moderate disturbances by weeding. Disturbance increased bryophyte richness and modulated treatment effects. In contrast to previous studies reporting indirect negative fertilization effects via increasing vascular plant productivity and reduced light levels, nitrogen fertilization directly reduced bryophyte cover and species richness, possibly because of toxic effects. Low vascular plant richness and dominance of fast-growing species reduced bryophyte richness. This might be because of decreased structural and resource niche heterogeneity in species-poor communities. Our results highlight novel mechanisms by which land-use intensification can affect bryophytes and suggest that a loss of vascular plant richness might have cascading effects on other taxonomic groups.

## Introduction

Grasslands cover about 40% of the earth’s terrestrial land surface ^1^. They harbor a high diversity of specialized taxa and may support the largest number of vascular plant species at small spatial scales (≤ 50 m^2^) ^2^. Grasslands have also suffered the highest rate of biodiversity loss over the past 100 years ^3^. In Western Europe, land-use intensification, which includes fertilizer application and frequent mowing or grazing with high stocking densities, is among the major threats ^4^. Nitrogen is the most frequently applied fertilizer and together with atmospheric deposition has become one of the major global change drivers. Although intensification increases grassland productivity, it simultaneously leads to a loss of biodiversity ^5^ and to a shift of the vascular plant community towards fast growth strategies, better adapted to nutrient-rich conditions ^6^. These changes in the vascular plant community might lead to small plants such as bryophytes being lost due to light competition ^7^. Bryophytes, comprising mosses, liverworts, and hornworts ^8^, can be an important part of the biodiversity of agricultural grasslands and can form a distinct layer on the soil surface below vascular plants ^9^. They further contribute to important ecosystem functions such as water and microclimate regulation and nutrient cycling ^10–12^. While changes in community composition following land-use intensification have been well studied for some taxa such as vascular plants, bryophytes have only rarely been considered. Understanding the mechanisms behind land-use effects on bryophytes is critical to promote restoration of bryophyte communities.

Nitrogen could affect bryophytes directly, but also indirectly via changing vascular plant community composition. Nitrogen can harm bryophytes directly due to: ammonium toxicity ^13,14^, osmotic effects, which can lead to browning of the tissues ^15^, and physiological processes, such as a disturbance of their carbon metabolism ^16^. But bryophytes can also benefit from nitrogen to a certain degree: studies have found that bryophyte growth increases up to moderate levels of fertilization ^17,18^. In addition to the direct effects, nitrogen fertilization can indirectly affect bryophytes in several ways. A major mechanism might be by changing vascular plant community composition and increasing vascular plant biomass ^19,20^. Higher vascular plant biomass, which is strongly positively related to the cover of vascular plants ^21,22^, leads to increased competition for light and space and the exclusion of less competitive, low-statured bryophytes from the understory ^23^. Although bryophytes have a low light compensation point ^24^ and can manage to grow with only short bursts of light ^25^, their short stature makes them dependent on sparse vegetation or patches of bare ground to receive enough sunlight and to have sufficient space for colonization ^26^. However, the relative importance of these direct fertilization effects compared to indirect effects remains largely unknown as only a few studies have so far tried to disentangle direct from indirect effects ^23,27^.

In addition to changes in vascular plant biomass, nitrogen could indirectly affect bryophytes by reducing vascular plant richness and shifting community composition towards fast growth. Observational studies have shown that bryophyte richness correlates positively with vascular plant richness ^28^, however, it is not clear whether vascular plant richness affects bryophyte cover and richness, or whether the two simply respond to a shared driver. Vascular plant richness could increase bryophyte abundance and richness by increasing the heterogeneity of resources, e.g. by providing a mosaic of different light levels or a larger range of soil pH, as has been found for temperate forests and vascular plants in the understory ^29^. In addition to potential positive effects, a high vascular plant richness could also decrease bryophyte cover and richness by leading to increased vascular plant biomass and cover ^30^. However, vascular plant richness could also increase bryophyte abundance and richness by increasing the heterogeneity of resources, e.g. by providing a mosaic of different light levels or a larger range of soil pH, as has been found for temperate forests and vascular plants in the understory ^29^. Experimental studies are needed to test this but the effect of vascular plant richness on bryophytes has only rarely been investigated in experiments manipulating vascular plant species number ^31,32^.

Changes in the functional composition of vascular plants following nitrogen enrichment could also influence bryophytes. A key axis of vascular plant functional composition variation is the leaf economics spectrum ^33,34^, distinguishing fast from slow growth strategy, which can be represented by traits such as specific leaf area (SLA) ^35^. Fast-growing communities – with a high mean SLA – are adapted to nutrient-rich conditions and might therefore produce more biomass, which in turn reduces light availability. However, fast vascular plant communities also have more rapidly decomposing litter and thus less organic material buildup on the ground ^36^, which might benefit bryophytes. Slow vascular plant species are well adapted to drought ^30^ and could thus provide a moister microclimate in summer which might facilitate bryophyte survival during dry periods. Because bryophytes take up nutrients over the whole surface area of their leaves, they react quickly to changes in their environments ^37^ and could be influenced by the conditions that fast- or slow-growing vascular plant communities produce. However, no studies have yet tested whether vascular plant fast-slow functional composition might affect bryophyte diversity and abundance in grasslands.

Changes in land use and vascular plant richness and composition also affect vascular plant consumers. Foliar fungal pathogens play an important role in grassland food webs and can sometimes have similar effects on vascular plant biomass as vertebrates ^38^. Foliar pathogens might therefore benefit bryophytes by reducing vascular plant dominance and increasing light levels. In agriculture, fungicides are used to protect crops and increase their yield. Copper-based and organic fungicides have been shown to directly reduce the germination of spores and asexual propagules in bryophytes, with long-term effects of copper-based fungicides due to their slow degradation and low water-solubility ^39^. However, the mechanism by which fungicides affect bryophytes in grasslands has not been studied so far. We hypothesized that fungicide will have an indirect negative effect on bryophytes, mediated by higher vascular plant cover following the removal of fungal pathogens.

Many grassland bryophyte species are adapted to low but regular disturbances, such as trampling by grazing animals ^40^, that create patches of bare ground to allow colonization and reduce competition for light with vascular plants ^41,42^. Moreover, bryophyte species might be separated by their growth form into two functional groups of acrocarpous and pleurocarpous species ^43^. The two groups have been proposed to being adapted to ecologically different habitat conditions ^44^ and therefore might respond differently to different land-use components in grasslands. Acrocarpous species need more open conditions than pleurocarpous. In biodiversity experiments, which investigate the effect of biodiversity on different taxa, plots are weeded manually to maintain diversity levels and community composition of vascular plants ^45^. Such weeding could be beneficial to bryophytes because it represents a type of disturbance ^46^ and would allow testing for the effect of disturbances in experiments ^32^ by comparing weeded plots with high soil disturbance and more open canopies, to unweeded plots with low soil disturbance and denser canopies.

In this study, we tested different mechanisms by which intensive land use can affect terricolous bryophytes. To do so, we recorded the number of species and the cover of bryophytes in weeded and unweeded subplots of a grassland experiment manipulating nitrogen fertilization, vascular plant species richness, vascular plant functional composition, and foliar fungal pathogen removal in a full-factorial design. Our hypotheses were:

- H1: Disturbance by weeding increases bryophyte cover and richness by producing bare ground for bryophyte colonization and increased light levels ^47^.
- H2: Nitrogen fertilization directly reduces bryophyte cover and richness via toxicity ^23,31,48^.
- H3: Nitrogen fertilization and fungicide application increase shading from vascular plants and decrease open space which in turn indirectly decreases bryophyte cover and richness ^19,49^.
- H4: Loss of vascular plant richness and shift to fast-growing species (high SLA) will reduce bryophyte species richness and cover due to lower structural heterogeneity ^29^ and changes in water and nutrient availability ^30^ respectively. Legacy effects of initial species richness and functional composition could persist for many years.

## Methods

### Study site

We conducted our study in the PaNDiv experiment, a large grassland field experiment in the Swiss lowlands (Münchenbuchsee, canton of Bern, 47°03’N, 7°46’E, 564 m a.s.l. ^36^). The annual mean temperature is 9.3 °C with a mean precipitation of 1022 mm (Federal Office of Meteorology and Climatology MeteoSwiss). Twenty different vascular plant species common to this grassland were sown at the beginning of the experiment in autumn 2015 (Table S1; nomenclature following Juillerat et al. 2017 ^50^). The bryophyte species have independently colonized the field since the establishment of the experiment.

### Experimental design

The experiment consists of 216 2 m × 2 m plots, separated by a 1 m grass path, arranged in 4 blocks of 54 plots each (see Figure S1). We applied four experimental treatments in a full-factorial design, manipulating: (1) the nitrogen availability (through fertilization), (2) the presence of fungal pathogens (through fungicide application), (3) the functional composition of the vascular plant community (represented here by community-weighted mean (CWM) SLA), and (4) the vascular plant richness (sowing of either 1, 4, 8 or 20 species). For this study, we selected 96 of the 216 plots from the initial design, which represents a balanced subsample containing 24 unique sets of species combinations (each replicated in four blocks) crossed with the nitrogen treatment and fungicide application. The subsample contains ten monocultures (five fast and five slow species), two combinations of 4- and 8-species mixtures for fast, mixed and slow species each, and two replicates of the 20-species plots. Fertilized plots received nitrogen as solid pellets of urea in April and in late June each year for a total of 100 kg N ha^−1^ y^−1^ (50 kg N ha^−1^ per application), corresponding to the amount of fertilizer proposed for mid-intensive to intensive grasslands of Switzerland ^51^. The fungicides Score Profi (24.8% difenoconazole; Syngenta Agro AG, Basel, Switzerland) and Ortiva (22.8% azoxystrobin; Syngenta Agro GmbH, Maintal, Germany) were applied each year four times during the growing period (0.2 mL of Score Profi and 0.4 mL of Ortiva mixed with 0.062 L of water per plot and time). Plots without fungicide application were sprayed with the same amount of water. Functional composition was manipulated by dividing the 20 sown species into ten fast- and ten slow-growing species (Table S1). Communities were then established with either only fast-, only slow-growing strategies, or both fast and slow strategies. To maintain the original sown vascular plant species richness and composition, one part of each plot (1.5 m × 2 m) was weeded, while the other part was left unweeded since the beginning of the experiment (0.5 m × 2 m). The weeding took place three times a year in April, July, and September. Having these two subsections on each plot enabled us to separate the effects of weeding on bryophyte cover and richness from the other treatment effects. The whole field was mown twice per year in mid-June and mid-August to simulate intermediate grassland management. Cut biomass was removed after mowing. For more information on the experimental setup see Pichon et al. ^36^.

### Vegetation sampling

From March to April 2023, we conducted a vegetation survey of the bryophyte species (all bryophytes were collected before the annual fertilization). We sampled all bryophyte species and visually estimated their cover in both the unweeded subplot and a part of the weeded subplot of the same size (0.5 m × 2 m). We collected bryophyte species that we were unable to identify in the field for later identification in the lab. The nomenclature of bryophytes follows Hodgetts et al. 2020 ^52^. For the subsequent analyses, we summed up the cover estimates of each species and species counts for total bryophyte cover and species richness values per subplot.

Additionally, we visually estimated the percentage cover of vascular plants in a summer campaign in August 2022 and a winter campaign in March/April 2023. We measured the summer cover of vascular plants because shading from vascular plants is likely to be most important when vascular plant biomass is high. Vascular plant winter cover may also be important as some bryophyte species (especially those from arable land) are short-lived and develop mostly from late summer on ^53^ and then grow throughout winter as long as temperatures remain above freezing. Open gaps in the vegetation cover can then be colonized. We therefore included both summer and winter cover of vascular plants in our initial models but finally excluded winter cover as it was never significant. In the unweeded subplots, the species richness of vascular plants had changed substantially over time and we therefore estimated the realized richness of these subplots in August 2022.

To calculate the functional composition of the vascular plant community, we measured the specific leaf area (SLA) of the 20 sown target species in monocultures in summer 2019, following the protocol from Garnier et al. ^54^. To account for species that occurred on the unweeded subplots and were not in the pool of 20 experimental species, we took SLA values from the “LEDA Traitbase” ^55^ and the “TRY Plant Trait Database” ^56^ (Table S2). For the TRY request, we first translated our species list to the nomenclature used in TRY to maximize the number of matches. We then merged the three separate SLA traits (Trait ID: 3115 petiole excluded, 3116 petiole included, 3117 undefined if petiole was included or not) to a compound SLA. SLA was available for all of the 51 species (except *Orobanche minor*, which has no leaves). SLA values for three species were not available in the TRY database, so we took the values from the LEDA database. We finally calculated the median value of SLA per species based on all database records and used the cover-weighted community mean per plot for further analysis (cover from August 2022).

### Statistical analyses

First, we tested the effect of weeding on bryophyte cover and richness by fitting linear mixed-effect models (LMMs) on all weeded and unweeded subplots (n = 192). We used nitrogen fertilization, fungicide application, weeding, and vascular plant cover as fixed effects. We fitted models with all three- and two-way interactions and without interactions. There were no significant interactions between weeding and the treatments, so we selected the model without any interactions. Block, plot number, and vascular plant combination were fitted as random effects to correct for correlations due to the experimental setup: the random effect for plot number accounts for the fact that the two subplots per plot are adjacent to each other and differ in the weeding but not in the manipulated treatments, while the combination (24 unique sets of species combinations) accounts for differences between particular combinations of vascular plant species and for the fact that each set of vascular plant species receives the four combinations of nitrogen and fungicide addition.

Second, we fitted LMMs to check for all two-way interactions between the manipulated treatments. We analyzed the weeded and unweeded subplots separately because the unweeded subplots have a different range in vascular plant richness and CWM SLA values than the weeded subplots. We fitted separate models with bryophyte cover and richness as response variables. The fixed effects were nitrogen fertilization, fungicide application, sown vascular plant richness, and sown CWM SLA, and the random effects were block and composition.

Third, we fitted structural equation models (SEMs) to determine whether the treatments affected the bryophytes directly, or indirectly through vascular plant cover, richness or functional composition. We fitted separate SEMs for the weeded and unweeded subplots and the structure of these differed due to the different disturbance regime. The SEM for the weeded subplots contained the treatments nitrogen fertilization, fungicide application, sown vascular plant richness, and sown vascular plant CWM SLA as exogenous variables. Vascular plant cover and bryophyte cover and richness were endogenous variables. We included a covariance between bryophyte cover and richness because we could not infer a directional effect between them. We did not include vascular plant realized richness and CWM SLA values in the models of the weeded subplots as they were very highly correlated with the sown metrics. For the unweeded subplots, the exogenous explanatory variables were the same and we fitted the realized vascular plant richness and realized CWM SLA as endogenous variables in addition to vascular plant cover. We fitted both the sown vascular plant richness and sown vascular plant CWM SLA to check whether there remained a historical effect of the sown vascular plant community. We expected the realized vascular plant richness and realized CWM SLA to be influenced by the manipulated treatments because they were not controlled by weeding. We did not account for the block effect as it was never significant in our LMMs. The full model that we tested is described in Figure S2 and all hypotheses for the conceptual model are included in Table S3.

Fourth, we analyzed bryophyte species composition in the weeded and unweeded subplots. We applied non-metric multidimensional scaling (NMDS) based on species abundance (cover values of the bryophytes) to check whether bryophyte species of different growth forms differed in their responses to the experimental treatments. For the NMDS we deleted taxa which were not unequivocal (mostly ‘cf.-species’ and specimens identified only to the genus level, Table S4). We split the bryophyte species into acrocarpous and pleurocarpous species and fitted LMMs to each of the two groups separately. The explanatory variables were nitrogen fertilization, fungicide application, weeding, and the cover of vascular plants. The response variables were a) cover of acrocarpous species, b) cover of pleurocarpous species, c) species richness of acrocarpous species, and d) species richness of pleurocarpous species.

All analyses were performed with R version 4.3.2 ^57^. We fitted the LMMs with the function ’lmer’ from the *lmerTest* package ^58^ and the SEMs with the function ’sem’ from the *lavaan* package ^59^. We fitted the NMDS with the function ‘metaMDS’ from the *vegan* package ^60^. We calculated estimates and confidence intervals using the function ’predict ‘ from the *effects* package ^61,62^.

## Results

Overall, we found between 32 (without uncertain identifications) and 45 different bryophyte species (Supplementary, Table S4). The average number of bryophyte species per subplot was 6.2 (range: 1 to 12). The average bryophyte cover per subplot was 3.21% (range: 0.02 to 33.63%). The most frequent species was *Ptychostomum rubens* (in 182 subplots or 95% of all subplots), followed by *Tortula acaulon* (141 subplots or 73% of all subplots) and *Dicranella staphylina* (139 subplots or 72% of all subplots). The species with the highest cover in a subplot was *Brachythecium rutabulum* (30%), followed by *Oxyrrhynchium hians* s.l. (12%) and *P. rubens* (3%). The most frequently occurring family was Bryaceae (relative frequency [= nr. of occurrences of species belonging to the family / total nr. of occurrences of all species] 38.4%), followed by Pottiaceae (rel. freq. 28.5%) and Brachytheciaceae (rel. freq. 15.6%).

**Fig. 1:**
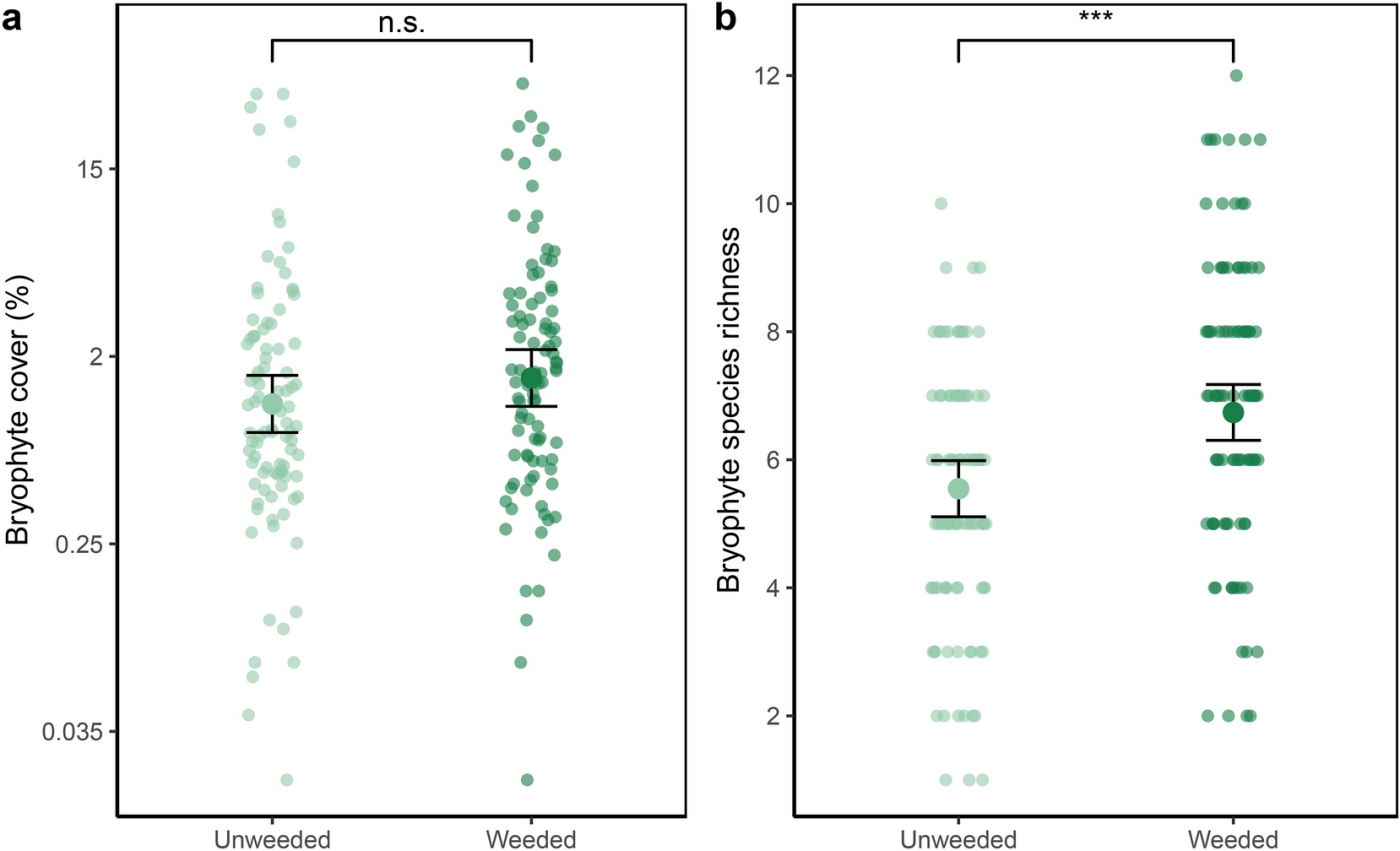
Effects of weeding on (**a**) bryophyte cover (%), and (**b**) bryophyte species richness per subplot (LMM, n = 192). The error bars represent 95% confidence intervals. Estimates and CI were generated with the effects package ^61,62^. n.s: not significant; ***: p < 0.001.

**Fig. 2:**
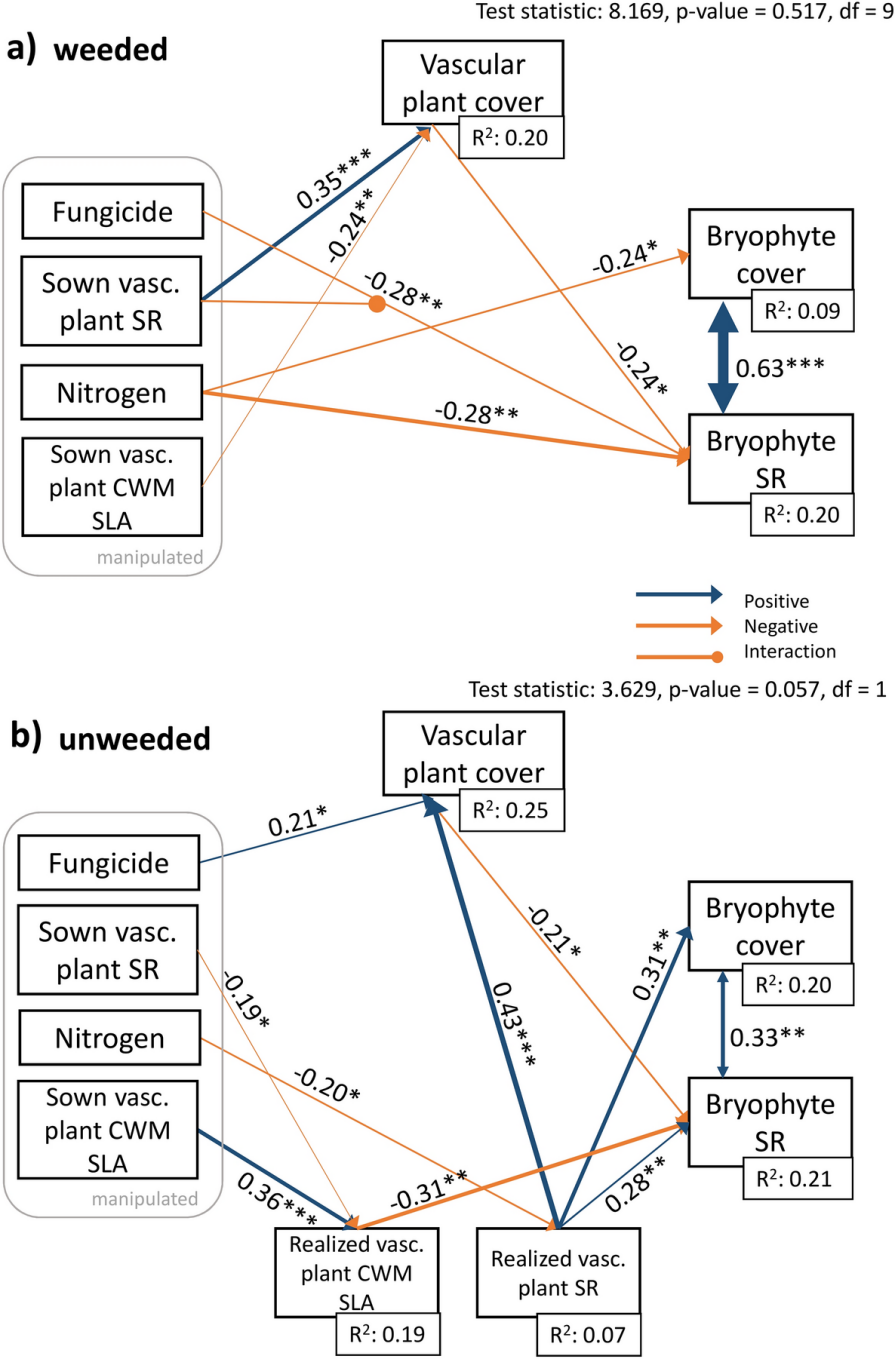
Effect of the manipulated treatments fungicide application, sown vascular plant species richness (SR), nitrogen fertilization, and sown vascular plant community-weighted mean (CWM) specific leaf area (SLA) on bryophyte cover and SR. Distinct structural equation models for the (**a**) weeded subplots (n = 96) and (**b**) unweeded subplots (n = 96). The unweeded subplots include the sown as well as the realized vascular plant SR and CWM SLA following invasion of non-sown species. Numbers adjacent to arrows show standardized path coefficients and the width of the line is proportional to the coefficient. Blue arrows indicate positive and orange arrows negative paths. Asterisks next to path coefficients indicate p-values: 0 < *** < 0.001 < ** < 0.01 < * < 0.05. Covariances are represented as two-headed arrows.

Weeding significantly increased bryophyte richness (p < 0.001) but not cover (p = 0.074; LMMs, n = 192, Fig. 1, model output: Tables S5 & S6 and Figures S3 & S4). In the weeded subplots vascular plant cover reduced bryophyte richness (SEM Fig. 2a, p = 0.036, n = 96).

**Fig. 3:**
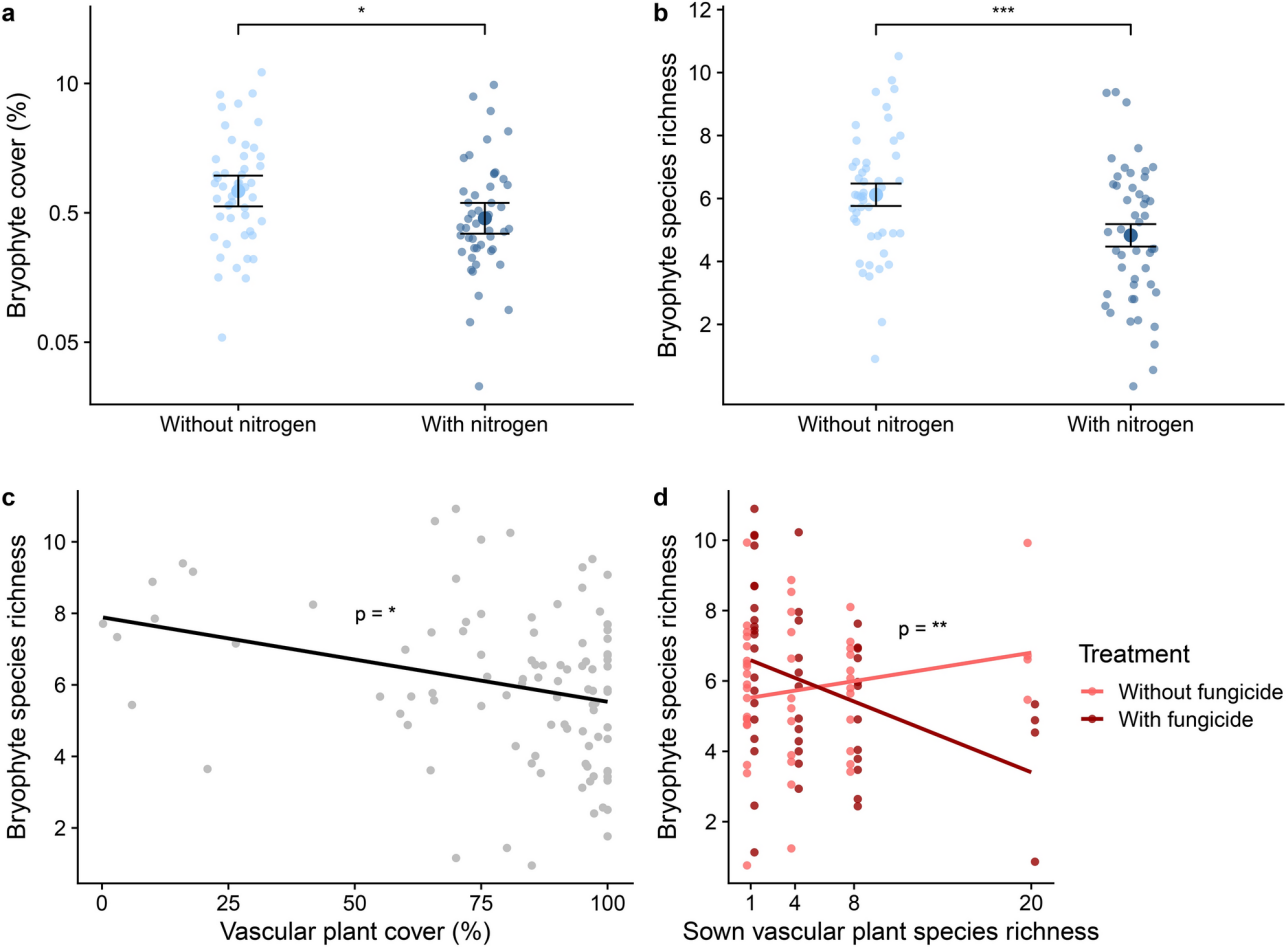
Partial plots from the structural equation model (SEM) on the **weeded** subplots displaying the partial effect of (**a**) nitrogen fertilization on bryophyte cover, (**b**) nitrogen fertilization on bryophyte species richness, (**c**) vascular plant cover on bryophyte species richness, and (**d**) sown vascular plant richness on bryophyte species richness depending on the fungicide treatment. The error bars (**a**&**b**) represent the confidence interval = 1.96*standard error. Raw data displayed as points. Asterisks next to path coefficients indicate p-values: 0 < *** < 0.001 < ** < 0.01 < * < 0.05.

In the weeded subplots, nitrogen fertilization directly reduced bryophyte cover and richness (SEM, Fig. 2a and partial plots Fig. 3a, b, p = 0.015 and 0.002, respectively, n = 96). Furthermore, sown vascular plant richness and sown vascular plant CWM SLA influenced bryophytes indirectly via vascular plant cover. Sown vascular plant richness increased vascular plant cover (Fig. 2a, p < 0.001), while sown vascular plant CWM SLA reduced it (Fig. 2a, p = 0.008). Vascular plant cover in turn reduced bryophyte species richness (Fig. 2a and 3c, p = 0.017). We found contrasting effects of fungicide on bryophyte richness depending on vascular plant richness: high sown vascular plant richness had a strong negative effect on bryophyte species richness when fungicides where applied, but a weak positive effect without fungicides (significant fungicide x vascular plant richness interaction; Fig. 3d, p = 0.007, n = 96).

**Fig. 4:**
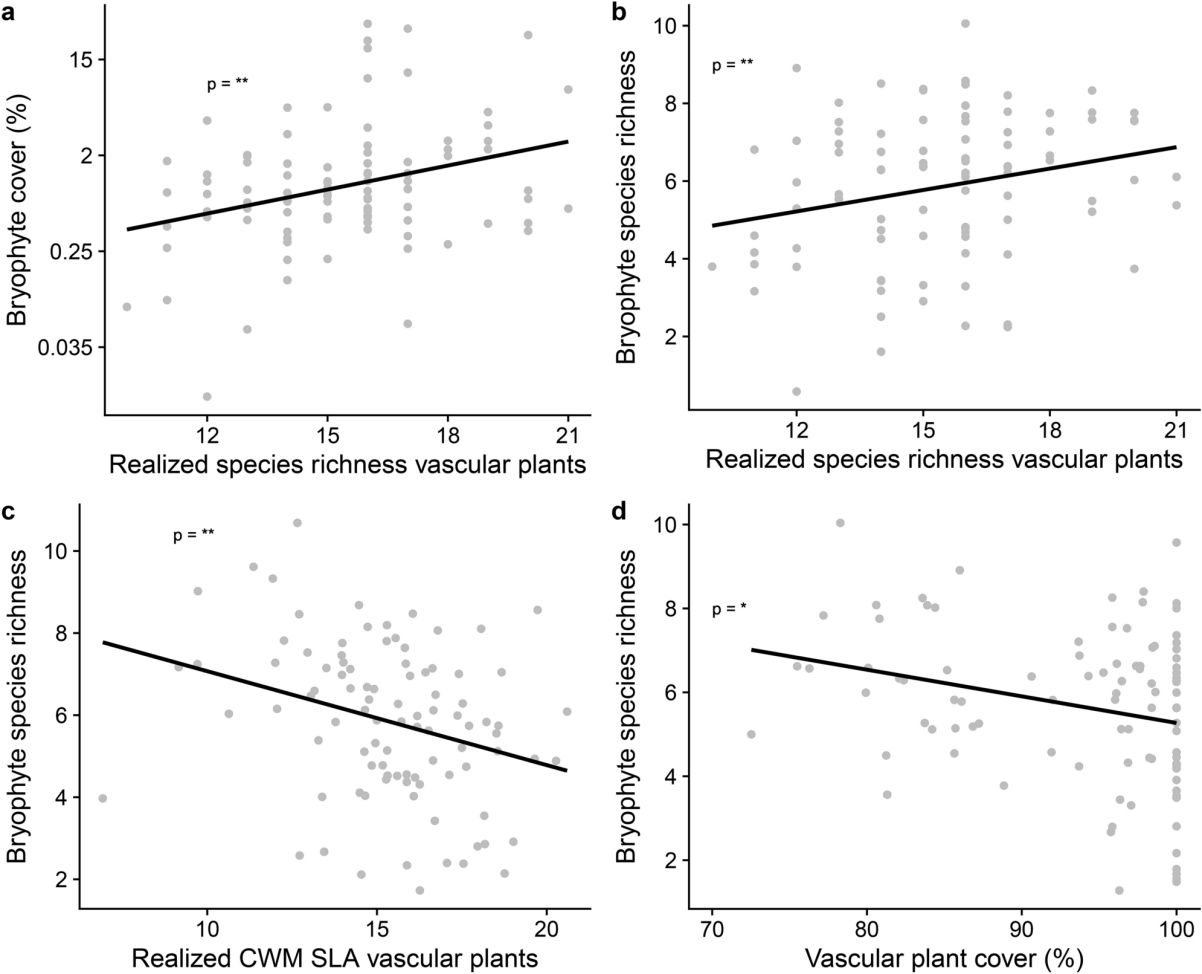
Partial plots from the structural equation model (SEM) on the unweeded subplots displaying the partial effect of (**a**) realized species richness of vascular plants on bryophyte cover, (**b**) realized species richness of vascular plants on bryophyte species richness, (**c**) realized vascular plant community-weighted mean (CWM) SLA on bryophyte species richness, and **d**) vascular plant cover on bryophyte species richness. Raw data displayed as points. Asterisks next to path coefficients indicate p-values: 0 < *** < 0.001 < ** < 0.01 < * < 0.05.

In the unweeded subplots, nitrogen had an indirect negative effect on bryophytes through realized vascular plant richness: nitrogen reduced realized vascular plant richness (Fig. 2b, p = 0.046, n = 96) which in turn increased bryophyte cover and richness (Fig. 2b, p = 0.003 and 0.009, n = 96). Realized vascular plant richness therefore had a direct positive effect on bryophyte species richness and cover. However, comparable to the weeded subplots, realized vascular plant richness also indirectly reduced bryophytes via its effect on vascular plant cover. Realized vascular plant richness increased vascular plant cover (Fig. 2b, p < 0.001) and vascular plant cover reduced bryophyte richness (Fig. 2b, p = 0.043), leading to a negative indirect effect of vascular plant richness. Furthermore, vascular plant cover increased with fungicide treatment (Fig. 2b, p = 0.016). Realized CWM SLA of vascular plants reduced bryophyte richness in the unweeded subplots (Fig. 2b and 4c; p = 0.003, n = 96). Realized CWM SLA of vascular plants was decreased by sown vascular plant richness (Fig. 2b, p = 0.050) and increased by sown CWM SLA of vascular plants (Fig. 2b, p < 0.001).

In both weeded and unweeded subplots there was a strong covariance between bryophyte cover and richness (Fig. 2a and b, p < 0.001 and p = 0.002 respectively).

Bryophyte response to the weeding treatment differed between the two growth forms. The pleurocarpous species and a part of the acrocarpous species reacted similarly to the treatments, while another part of acrocarpous species benefited more from weeding treatment (NMDS, Fig. S5). Weeding increased the richness of both groups (acrocarpous: LMM Table S7c, n = 189, p = 0.002, pleurocarpous: LMM Table S7d, n = 189, p = 0.001), and the cover of pleurocarpous species (LMM Table S7b, n = 146, p = 0.011). However, vascular plant cover reduced the cover (LMM Table S7a, n = 187, p < 0.001) and richness of acrocarpous species (LMM Table S7c, n = 189, p < 0.001), while it increased the cover of pleurocarpous species (LMM Table S7b, n = 146, p = 0.026).

## Discussion

This is the first study to experimentally test the effect of several aspects of land-use change on bryophytes for sites differing in disturbance. Our results highlight that diverse vascular plant communities dominated by slow species support a greater abundance and richness of bryophytes. We found that regular disturbance through weeding was a major driver of bryophyte communities and increased bryophyte species richness, supporting our first hypothesis. Weeding strongly reduced vascular plant cover. In our study the minimum cover was 72.53 % in the unweeded subplots but was as low as 0.20 % in the weeded subplots (mean weeded subplots = 80 %, unweeded = 94 %, Figure S6). In natural grasslands vascular plant cover may also be decreased by disturbances, for example by grazing, or mice or earthworm activity ^63^, and bryophyte species from agricultural land are dependent on these disturbances ^64^. In this regard, weeded subplots represent frequently disturbed habitats with open canopies and a high proportion of bare ground. In these habitats the effect of bare ground seemed to be more important in shaping the bryophyte community. Unweeded subplots represent more established communities typical of semi-natural grasslands with regular mowing, and they have closed canopies and a lower proportion of bare ground. The positive effect of weeding is in line with another one of our results, namely that vascular plant cover decreased bryophyte richness in the weeded subplots (only marginally significant in the unweeded subplots), a result that has been found by previous observational and experimental studies ^49,65,66^. Patches of bare ground are of major importance for many bryopohytes, but in particular for short-lived and acrocarpous bryophyte species, which are mainly small species, weak competitors but fast colonizers ^67^. A high proportion of the species we found were acrocarpous mosses of the families *Pottiaceae* and *Bryaceae*, which are characterized by a high frequency of sexual and/or asexual propagules ^68^. Particularly, the species from the genus *Bryum* s.l. (*Bryum, Ptychostomum*) and *Dicranella staphylina* produce many rhizoidal gemmae enabling them to persist in the diaspore bank and quickly colonize newly opened space when favorable habitat conditions reappear ^32,41,64,69^. In line with previous studies ^70,71^, our findings highlight the importance of moderate soil disturbances, that create gaps for the colonization of less competitive taxa, and at the same time can reduce the dominance of vascular plant species.

In accordance with the second hypothesis we found that nitrogen fertilization directly reduced bryophyte cover and richness in the weeded subplots, which suggests a toxic effect on bryophyte tissue. Previous studies have shown that bryophytes tend to have unimodal growth responses to increasing fertilizer amounts and therefore can tolerate moderate fertilization ^17,18^. However, the levels of fertilization used in these studies were much lower than the levels usually used in agricultural systems and in our experiment (100 kg N ha ^-1^ y ^-1^). For instance, Dirkse and Martakis ^17^ reported negative responses of some sensitive species at doses of 30 kg N ha^-1^ y ^-1^ and a decline of tolerant species such as tall-growing *Brachythecium* species at doses larger than 60 kg N ha ^-1^ y ^-1^. Our results of a direct negative fertilization effect agree with toxic nitrogen effects reported by others. For instance, Boch et al. ^23^ found direct negative fertilizer effects, decreasing bryophyte cover and richness in Swiss mountain grasslands. In their experiment, the fertilizer amounts were also lower than in our experiment as they were adapted to mountain grassland land use. A toxic effect has also been hypothesized by Haworth et al. ^72^ in calcareous grasslands in the UK in relation to soil acidification after nitrogen fertilization. The authors found that bryophytes declined even though vascular plant cover did not change in response to nitrogen fertilization (with nitrate or sulfate) and hence assumed toxic effects on the bryophytes’ metabolic processes. Other studies support the idea that solid fertilizers cause the drying out of bryophyte tissue due to an osmotic effect, so-called “browning” ^15,23^. The toxicity of nitrogen is probably due to the formation of ammonium after fertilizer application ^13,14,73^. This can lead to physiological changes in bryophytes, which can disturb the cellular carbon metabolism ^16,48^. We only found the toxic effect in the weeded subplots, which had a markedly lower proportion of vascular plant cover. This might be an indication that toxicity plays a role when the solid fertilizer is in direct contact with the bryophytes. In contrast to our expectation ^9,20,31,48,74,75^, we did not find an indirect effect of nitrogen on bryophytes via vascular plant cover (reject hypothesis three). The reason for this missing relationship is different for the weeded and unweeded subplots. The vascular plant cover in the weeded subplots is mainly determined by the manual weeding that removes weeds. In the unweeded subplots there might not have been an indirect effect of nitrogen because the cover of vascular plants is high irrespective of the nitrogen treatment (Fig. S6).

We found opposite effects of vascular plant richness: a negative indirect effect through vascular plant cover, as expected due to light competition, but also a positive direct effect (support hypothesis four). In the weeded subplots, vascular plant richness increased bryophyte species richness in the absence of fungicide, while in the unweeded subplots, realized vascular plant richness increased bryophyte cover and richness. The positive effect of vascular plant richness might be because a high species richness of vascular plants ensures continuous but moderate shading during the driest season that can improve the microclimate for bryophytes below the canopy ^76^. Diverse communities of vascular plants have been shown to lower temperatures at the soil surface, lower the vapor pressure deficit, and increase surface soil moisture ^77^. Furthermore, vascular plant richness could benefit bryophytes by creating a more structurally heterogenous habitat and providing more different light niches, i.e. some open areas and some more shaded areas. In forest ecosystems it is known that a diverse tree community increases the structural complexity of the stand ^78,79^. Moreover, Helbach et al. ^29^ have shown that increased structural complexity of tree stands increases the heterogeneity of light and soil resources below the canopy. They also found that high levels of heterogeneity increase the species richness of herbs in the understory. These results might be transferrable to our study system in grasslands where bryophytes form the understory and are influenced by the structural composition of the vascular plant canopy. In line with another study ^80^, we also found that nitrogen reduced realized vascular plant richness and thus indirectly reduced bryophyte cover and species richness. Interestingly, vascular plant richness indirectly reduced bryophyte cover in the weeded subplots by increasing vascular plant cover. This shows the importance of accounting for disturbance effects in biodiversity experiments, without which we would have wrongly inferred a negative effect of vascular plant richness on bryophytes. A false negative relationship would appear because plots with low species richness of vascular plants are more invaded by other vascular plants ^81,82^ and require higher levels of weeding to maintain diversity levels compared to more diverse plots. We found the effects of realized species richness of vascular plants only in the unweeded subplots, perhaps because of the relatively closed vascular plant canopy compared to the weeded subplots. Furthermore, there were big differences in the distribution of the values of realized vascular plant richness (Fig. S7). The mean in the weeded subplots was four species, while it was 15 in the unweeded subplots (range weeded subplots: 1 to 16 species, range unweeded subplots: 10 to 21 species). Because the values are strongly controlled by the weeding, we might have only found an effect of vascular plant richness in the unweeded subplots, where we have a natural range and distribution of values. Our results show that losses of vascular plant richness might have cascading effects on bryophytes leading to further extinctions, which highlights the importance of restoring vascular plant richness in grasslands.

We also found that vascular plant fast-slow functional composition affected bryophytes (support hypothesis four). In the unweeded subplots, high vascular plant CWM SLA directly reduced bryophyte richness, indicating that communities dominated by fast-growing vascular plant species supported fewer bryophyte species. To our knowledge this is the first study showing that changes in vascular plant resource-use traits can affect bryophytes. Our results might be explained by different mechanisms: fast-growing vascular plants may have a high initial growth early in the season which allows them to maintain dominance in the community ^83^. Fast vascular plants may therefore shade bryophytes earlier, compared to the slow vascular plants and this early shading could have an effect that is not reflected in our measure of cover. On the other hand, species with low SLA are more drought-tolerant and there is more water available in plots dominated by slow-growing species ^30^. Thus slow-growing species do not dry out as fast in summer and this might benefit bryophytes because the cover of the slow vascular plants is high enough to maintain moist conditions at the soil level for a longer time period. Alternatively, the effect of SLA could be mediated by the litter quantity and quality (nutrient content) of the vascular plant community. Despite the common assumption of a negative effect of litter covering bryophytes and thereby increased shading, there is evidence that litter can also favor bryophytes. Studies have found that a certain amount of litter can be beneficial at least to some pleurocarpous bryophytes because it stores water and represents a source of nutrients ^84,85^. The litter of slow-growing vascular plants (low SLA) decomposes slower ^36^ and thereby provides moderate cover of the bryophyte layer. This could represent a reason for the positive effect of slow-growing vascular plants. Lastly, species with a low SLA might represent an optimal trade-off between moisture requirement and shade production for bryophytes, similar to the result of Fergus et al. ^32^ where species richness of grasses increased while richness of legumes decreased bryophyte richness. A vascular plant composition shift following nitrogen fertilization will further negatively affect bryophytes.

Fungicide also affected bryophytes through two mechanisms. Reducing fungal pathogens with fungicide indirectly reduced bryophyte species richness by increasing vascular plant cover on the unweeded subplots. This matches our expectation, as fungicide removes fungal pathogens, and increases vascular plant cover, which creates more shade. The reason why we only found this effect in the unweeded subplots could be because there is a higher percentage of vascular plant cover present for the fungicides to act on compared to the weeded subplots. In addition, fungicide application reversed the effect of vascular plant richness on bryophyte species richness in the weeded subplots. This was because fungicide strongly reduced bryophyte richness in plots with high sown vascular plant richness, while in low diversity plots fungicide had hardly any effect on bryophyte species richness. It is possible that fungicide leads to an increased dominance of fast-growing plants ^86^ which support fewer bryophyte species, and that this effect is strongest in the 20 species plots where there was the greatest opportunity for plant composition to shift. We also cannot exclude the possibility that the negative effect of fungicide in the diverse plots is due to the composition of the fungicide solution: non-soluble fungicides may form a layer on the leaves of the bryophytes and reduce gas exchange. Furthermore, greenhouse experiments have shown that fungicides inhibit the formation of bryophyte sporophytes and asexual propagules ^39^. However, these direct toxic effects would not be expected to be stronger in species rich plots. Our results may therefore suggest that pathogens play a role, not only in maintaining vascular plant diversity but also in indirectly promoting the diversity of bryophytes.

Another reason for the differing responses of the bryophytes in the weeded and unweeded subplots might be differences in the species composition related to growth forms of bryophytes. In general, both growth forms benefitted from weeding but the group of acrocarpous species benefitted even more than the pleurocarpous group. This was also reflected in the negative effect of vascular plant cover on the cover and richness of acrocarpous species. Acrocarpous species that grow as small cushions, are adapted to microsites with good light conditions and low competition, e.g. open grassland patches ^44^. They are proposed to better survive hot and dry conditions at low vascular plant cover because of a higher resistance to water loss. Moreover, acrocarpous species are often short-lived, and might survive unfavorable periods in the forms of gemmae or spores ^44^. In contrast, pleurocarpous species cover larger areas due to their growth form and can better deal with the shady conditions and moderate litter cover below the vascular plant canopy ^44,87,88^.

## Conclusions

Our study shows that the relationships between vascular plants and bryophytes are complex, highlighting the need for studies mechanistically exploring these patterns. Our study illustrates that structural equation modeling enables more mechanistic explorations of the complex responses of biodiversity patterns to changed environmental variables. Neglecting indirect effects of habitat changes following nutrient enrichment, such as increased vascular plant cover, might even lead to incomplete conclusions and could compromise management recommendations, as these effects are sometimes more important than direct effects. From an applied point of view, our findings that bryophytes are negatively affected by nitrogen fertilization, by the loss of vascular plant richness, and by increased CWM SLA, highlight several mechanisms by which land-use intensification might threaten bryophyte communities. This shows the need to reduce fertilizer input in agricultural grasslands to maintain biodiversity, not just of vascular plants but of associated groups such as bryophytes. However, we also showed that moderate soil disturbances, which might be also actively applied in grasslands e.g. by mechanically harrowing a meadow, might enhance bryophyte diversity.

## Supplementary Information

Supplementary Information.

## Data Availability

The datasets generated and analyzed during the current study are available in the EnviDat repository, https://envidat.ch/#/metadata/data-on-bryophyte-diversity-in-the-pandiv-experiment.
