# Supplementary material for: Nitrogen enrichment and vascular plant richness loss reduce bryophyte richness
Source: Sci Rep. 2025 Feb 3;15:4049. doi: 10.1038/s41598-025-88425-2 (PMC11790842; doi:10.1038/s41598-025-88425-2)
Supplement: Supplementary file 1 — Supplementary Information. [file 41598_2025_88425_MOESM1_ESM.docx]

# **Nitrogen enrichment and vascular plant richness loss reduce bryophyte richness**

Maeve Lin ^1*^, Ariel Bergamini ^1^, Noémie A. Pichon ^1^, Eric Allan ^2,3,4^, Steffen Boch ^1^

# Supplementary

**Figure S1**: The experimental site (PaNDiv) in Münchenbuchsee, BE. Including map of Switzerland with location and sampling design on the plot-level. Photo credits: Hugo Vincent. Map generated with R version 4.3.2 [1], package *terra* [2], Shapefiles from the Federal Office of Topography swisstopo.


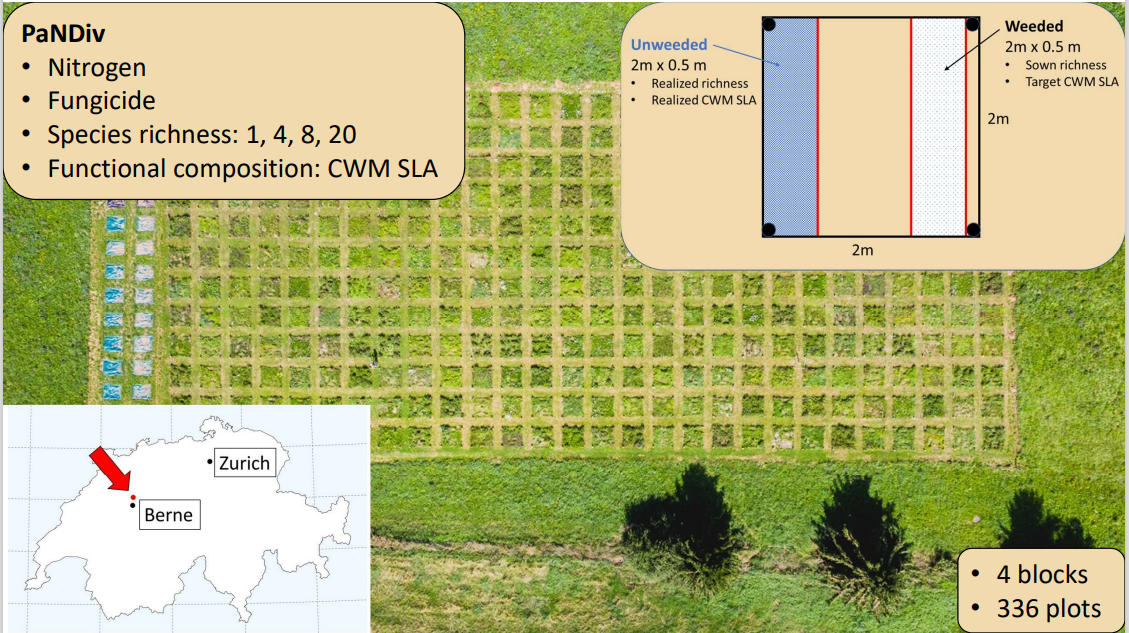


**Table S1.** PaNDiv experimental vascular plant species. All species are perennial. Nomenclature follows Juillerat et al. 2017 [3].

| Species | Resource economics | Group | SLA [mm^2^/mg] |
| --- | --- | --- | --- |
| *Dactylis glomerata* | fast | grass | 25.29 |
| *Holcus lanatus* | fast | grass | 23.18 |
| *Lolium perenne* | fast | grass | 18.22 |
| *Poa trivialis* | fast | grass | 36.31 |
| *Anthriscus sylvestris* | fast | herb | 20.59 |
| *Crepis biennis* | fast | herb | 18.57 |
| *Galium album* | fast | herb | 26.69 |
| *Heracleum sphondylium* | fast | herb | 16.55 |
| *Rumex acetosa* | fast | herb | 24.11 |
| *Taraxacum officinale aggr.* | fast | herb | 15.74 |
| *Anthoxanthum odoratum* | slow | grass | 25.47 |
| *Bromus erectus* | slow | grass | 18.48 |
| *Festuca rubra* | slow | grass | 17.94 |
| *Helictotrichon pubescens* | slow | grass | 20.83 |
| *Achillea millefolium* | slow | herb | 11.79 |
| *Centaurea jacea* | slow | herb | 19.27 |
| *Daucus carota* | slow | herb | 14.92 |
| *Plantago media* | slow | herb | 16.06 |
| *Prunella grandiflora* | slow | herb | 20.27 |
| *Salvia pratensis* | slow | herb | 19.30 |

**Table S2**. Database-derived SLA values for the spontaneously growing (non-sown) vascular plant species growing in the unweeded subplots. Nomenclature follows Juillerat et al. 2017 [3].

| Species | SLA [mm^2^/mg] |
| --- | --- |
| *Arrhenatherum elatius* | 29.37 |
| *Bellis perennis* | 28.70 |
| *Briza media* | 21.50 |
| *Bromus hordeaceus* | 27.74 |
| *Capsella bursa-pastoris* | 29.50 |
| *Centaurea scabiosa* | 19.68 |
| *Cerastium fontanum* | 29.40 |
| *Chenopodium album* | 25.75 |
| *Cirsium arvense* | 14.16 |
| *Dianthus carthusianorum* | 17.89 |
| *Erigeron annuus* | 21.93 |
| *Fallopia convulus* | 25.40 |
| *Festuca arundinacea* | 18.10 |
| *Festuca pratensis* | 23.29 |
| *Geranium dissectum* | 25.60 |
| *Geranium molle* | 25.60 |
| *Glechoma hederacea* | 36.46 |
| *Knautia arvensis* | 18.83 |
| *Lamium purpureum* | 32.30 |
| *Lathyrus pratensis* | 25.96 |
| *Leontodon hispidus* | 22.96 |
| *Leucanthemum vulgare* | 18.34 |
| *Lolium multiflorum* | 28.90 |
| *Lotus corniculatus* | 22.89 |
| *Medicago lupulina* | 27.90 |
| *Myosotis arvensis* | 28.80 |
| *Onobrychis viciifolia* | 18.33 |
| *Orobanche minor* | NA |
| *Pilosella officinarum* | 20.40 |
| *Plantago lanceolata* | 19.52 |
| *Poa annua* | 34.80 |
| *Poa pratensis* | 22.22 |
| *Polygonum aviculare* | 29.70 |
| *Ranunculus acris* | 22.50 |
| *Rumex obtusifolius* | 20.09 |
| *Sanguisorba minor* | 17.70 |
| *Scabiosa columbaria* | 20.06 |
| *Silene dioica* | 41.54 |
| *Silene nutans* | 21.20 |
| *Silene vulgaris* | 19.80 |
| *Sonchus oleraceus* | 22.07 |
| *Stellaria media* | 47.00 |
| *Tragopogon pratensis* | 18.20 |
| *Trifolium dubium* | 25.10 |
| *Trifolium pratense* | 21.93 |
| *Trifolium repens* | 31.18 |
| *Trisetum flavescens* | 22.60 |
| *Veronica arvensis* | 26.00 |
| *Veronica filiformis* | 37.65 |
| *Veronica persica* | 42.30 |
| *Vicia sepium* | 37.61 |

**Figure S2: Conceptual SEM**


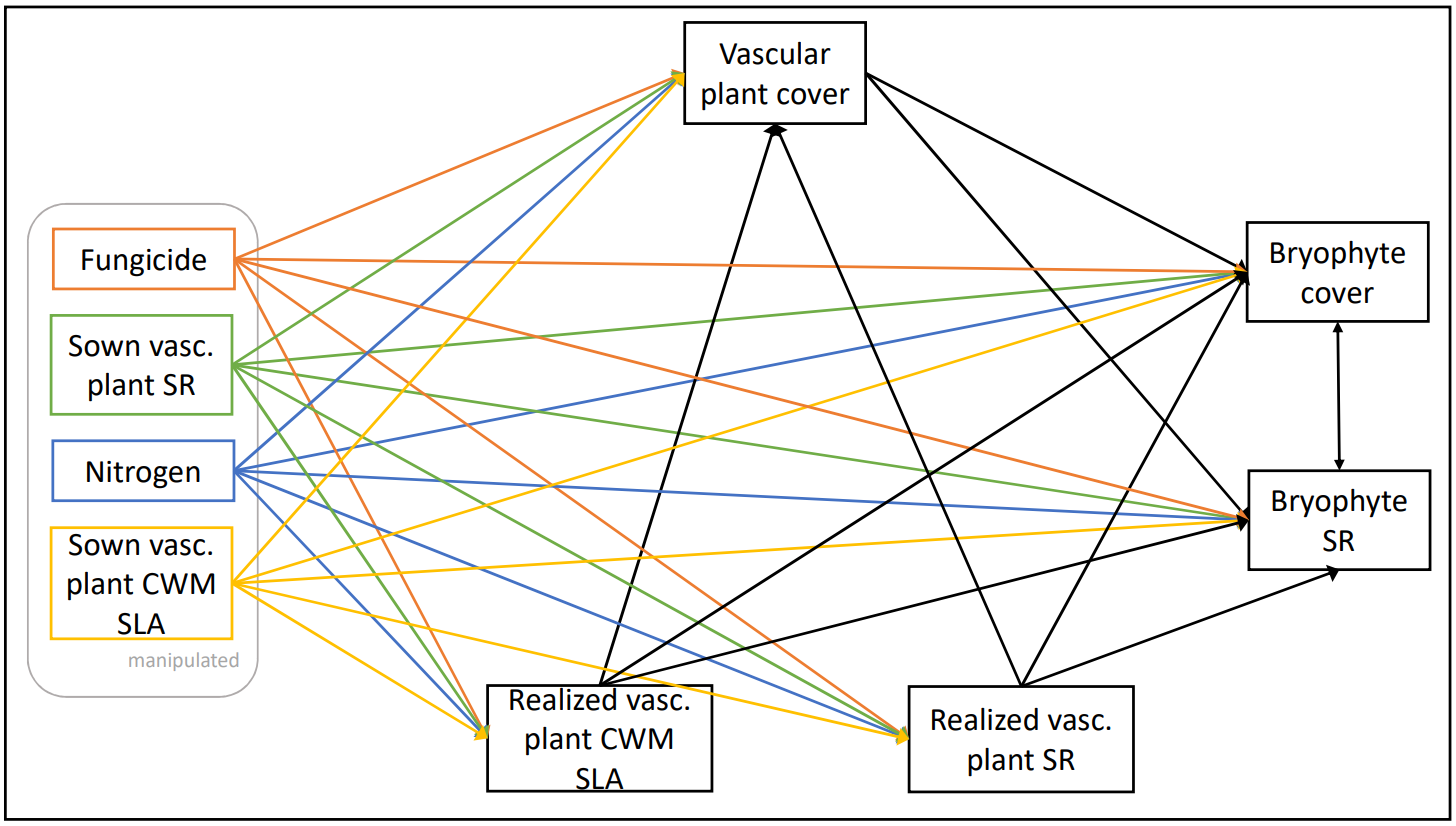


**Figure S2.** The full structural equation model (SEM) that was used in the analysis. We tested all paths from the manipulated treatments (left box) to the measured variables. Single headed arrows represent paths and double headed arrows covariances (only between Bryophyte cover and Bryophyte SR). They are included in the model to account for correlations between variables where we could not infer a directional effect. The paths are explained in Table S3. CWM: community weighted mean; SLA: specific leaf-area; SR: species richness.

**Table S3.** Hypothesized mechanisms for the paths in the structural equation model (Figure S2). The direction of the effects of the hypothesized mechanisms is given in brackets. See introduction for further explanations to the hypothesized mechanisms.

| Path | Hypothesized mechanism | References |
| --- | --- | --- |
| Fungicide 🡪 bryophyte cover | (-) inhibits germination of spores and/or asexual propagules | Oesau et al. [4] |
| Fungicide 🡪 bryophyte SR | (-) inhibits germination of spores and/or asexual propagules | Oesau et al. [4] |
| Fungicide 🡪 plant cover | (+) pathogen release | Cappelli et al.[5] |
| Fungicide 🡪 realized plant SLA | (+) growth-defense trade-off | Pichon et al. [6] |
| Fungicide 🡪 realized plant SR | (-) | - |
| Plant SR 🡪 bryophyte cover | (+) increased structural heterogeneity | Helbach et al. [7] |
| Plant SR 🡪 bryophyte SR | (+) increased structural heterogeneity | Helbach et al. [7] |
| Plant SR 🡪 plant cover | (+) positive diversity-productivity relationship | Pichon et al., Walde et al. [6,8] |
| Plant SR 🡪 realized plant SLA | (-) | Pichon et al., Cappelli et al. [6,9] |
| Plant SR 🡪 realized plant SR | (+) |  |
| Nitrogen 🡪 bryophyte cover | (-) toxicity | Arróniz-Crespo et al.[10] |
| Nitrogen 🡪 bryophyte SR | (-) toxicity of ammonium | ​​Boch, Allan, et al. [11] |
| Nitrogen 🡪 plant cover | (+) no nitrogen-limitation increases growth | ​​Bergamini & Pauli; Pichon & Cappelli; Walde et al. [8,12,13]​ |
| Nitrogen 🡪 plant SLA | (+) fast-growing plants outcompete slow-growing | ​​Pichon et al.; Pichon & Cappelli​ [6,14] |
| Nitrogen 🡪 plant SR | (-) increase in light competition | ​​Mitchell et al.; Stevens et al. [15,16] |
| Plant SLA 🡪 bryophyte cover | (+) drought-tolerance of slow-growing plants | Walde et al. [8] |
| Plant SLA 🡪 bryophyte SR | (+) drought-tolerance of slow-growing plants | Walde et al. [8] |
| Plant SLA 🡪 plant cover | (+) fast-growing plants cover more area | Cappelli et al. [5] |
| Plant SLA 🡪 realized plant SLA | (+) | - |
| Plant SLA 🡪 realized plant SR | No clear hypothesis | - |
| Plant cover 🡪 bryophyte cover | (-) light competition | Ochoa-Hueso et al. [17] |
| Plant cover 🡪 bryophyte SR | (-) light competition | Löbel et al. [18] |

**Table S4.** List of identified bryophyte species, their family, the number of the 192 studied subplots they were found in and their frequency (calculated as [(no. of subplots / total no. of subplots) * 100]). Marked in red are the taxa that were removed to compute the NMDS. Nomenclature follows Hodgetts et al. [19]. Growth form according to Meusel [20].

| Species | Family | Growth form | Number of subplots | Frequency [%] |
| --- | --- | --- | --- | --- |
| *Amblystegium serpens* | *Amblystegiaceae* | pleurocarpous | 10 | 5.21 |
| *Barbula unguiculata* | *Pottiaceae* | acrocarpous | 44 | 22.92 |
| *Brachytheciastrum velutinum* | *Brachytheciaceae* | pleurocarpous | 1 | 0.52 |
| *Brachythecium rutabulum* | *Brachytheciaceae* | pleurocarpous | 129 | 67.19 |
| *Brachythecium* cf. *rutabulum* | *Brachytheciaceae* | pleurocarpous | 1 | 0.52 |
| cf. *Brachythecium rutabulum* | *Brachytheciaceae* | pleurocarpous | 3 | 1.56 |
| *Bryum argenteum* | *Bryaceae* | acrocarpous | 62 | 32.29 |
| *Bryum dichotomum* | *Bryaceae* | acrocarpous | 22 | 11.46 |
| *Bryum* cf. *dichotomum* | *Bryaceae* | acrocarpous | 2 | 1.04 |
| *Bryum klinggraeffii* | *Bryaceae* | acrocarpous | 53 | 27.60 |
| *Bryum* cf. *klinggraeffii* | *Bryaceae* | acrocarpous | 1 | 0.52 |
| *Bryum radiculosum* | *Bryaceae* | acrocarpous | 1 | 0.52 |
| *Bryum ruderale* | *Bryaceae* | acrocarpous | 48 | 25.00 |
| *Bryum* spec. | *Bryaceae* | acrocarpous | 28 | 14.58 |
| *Bryum violaceum* | *Bryaceae* | acrocarpous | 45 | 23.44 |
| *Bryum* cf. *violaceum* | *Bryaceae* | acrocarpous | 5 | 2.60 |
| *Calliergonella cuspidata* | *Pylaisiaceae* | pleurocarpous | 11 | 5.73 |
| *Ceratodon purpureus subsp. purpureus* | *Ditrichaceae* | acrocarpous | 24 | 12.50 |
| *Dicranella staphylina* | *Dicranaceae* | acrocarpous | 139 | 72.40 |
| *Didymodon* cf. *acutus* | *Pottiaceae* | acrocarpous | 1 | 0.52 |
| *Didymodon* cf. *ferrugineus* | *Pottiaceae* | acrocarpous | 1 | 0.52 |
| *Funaria hygrometrica* | *Funariaceae* | acrocarpous | 4 | 2.08 |
| *Hypnum cupressiforme* | *Hypnaceae* | pleurocarpous | 6 | 3.13 |
| *Hypnum* cf. *cupressiforme* | *Hypnaceae* | pleurocarpous | 1 | 0.52 |
| *Kindbergia praelonga* | *Brachytheciaceae* | pleurocarpous | 1 | 0.52 |
| *Oxyrrhynchium hians* s.l. | *Brachytheciaceae* | pleurocarpous | 48 | 25.00 |
| *Physcomitrium pyriforme* | *Funariaceae* | acrocarpous | 6 | 3.13 |
| cf. *Physcomitrium* | *Funariaceae* | acrocarpous | 1 | 0.52 |
| *Pottia/Phascum* spec. | *Pottiaceae* | acrocarpous | 26 | 13.54 |
| *Pottiaceae* spec. | *Pottiaceae* | acrocarpous | 1 | 0.52 |
| *Pseudocrossidium hornschuchianum* | *Pottiaceae* | acrocarpous | 3 | 1.56 |
| *Ptychostomum* cf. *creberrimum* | *Bryaceae* | acrocarpous | 1 | 0.52 |
| *Ptychostomum* cf. *imbricatulum* | *Bryaceae* | acrocarpous | 1 | 0.52 |
| *Ptychostomum imbricatulum* | *Bryaceae* | acrocarpous | 2 | 1.04 |
| *Ptychostomum rubens* | *Bryaceae* | acrocarpous | 182 | 94.79 |
| *Pylaisia/Platygyrium* spec. | *Pylaisiaceae* | pleurocarpous | 1 | 0.52 |
| *Rhynchostegium megapolitanum* | *Brachytheciaceae* | pleurocarpous | 1 | 0.52 |
| *Riccia sorocarpa* | *Ricciaceae* | NA | 1 | 0.52 |
| *Schistidium apocarpum* | *Grimmiaceae* | acrocarpous | 2 | 1.04 |
| *Streblotrichum convolutum* | *Pottiaceae* | acrocarpous | 109 | 56.77 |
| *Tortula acaulon* | *Pottiaceae* | acrocarpous | 141 | 73.44 |
| *Tortula* cf. *acaulon* | *Pottiaceae* | acrocarpous | 1 | 0.52 |
| *Tortula muralis* subsp. *muralis* | *Pottiaceae* | acrocarpous | 5 | 2.60 |
| *Weissia longifolia* | *Pottiaceae* | acrocarpous | 1 | 0.52 |
| *Weissia* spec. | *Pottiaceae* | acrocarpous | 4 | 2.08 |

**Table S5.** Summary of the linear mixed-effect model on the effect of nitrogen fertilization, fungicide application, weeding, and cover of vascular plants (scaled) on bryophyte species richness. Plot number, block number, and the composition of vascular plants of each plot were fitted as random factors. Nitrogen, fungicide, and weeding were coded as factors. Bold font indicates statistical significance (at α = 0.05).

| Fixed effects | Estimate | DF | SE | p |
| --- | --- | --- | --- | --- |
| (Intercept) | -0.02 | 10.89 | 0.17 | 0.891 |
| Nitrogen | -0.42 | 90.09 | 0.15 | **0.006** |
| Fungicide | -0.03 | 90.61 | 0.15 | 0.815 |
| Weeding | 0.50 | 103.20 | 0.12 | **< 0.001** |
| Cover vascular plants (scaled) | -0.26 | 175.87 | 0.07 | **< 0.001** |

**Table S6.** Summary of the linear mixed-effect model on the effect nitrogen fertilization, fungicide application, weeding, and cover of vascular plants (scaled) on log (**bryophyte cover**). Block number, plot number, and the composition of vascular plants of each plot were fitted as random factors. Nitrogen, fungicide, and weeding were coded as factors. Bold font indicates statistical significance (at α = 0.05).

| Fixed effects | Estimate | DF | SE | p |
| --- | --- | --- | --- | --- |
| (Intercept) | 0.29 | 14.97 | 0.18 | 0.128 |
| Nitrogen | -0.44 | 66.45 | 0.16 | **0.008** |
| Fungicide | -0.32 | 66.78 | 0.16 | 0.048 |
| Weeding | 0.19 | 101.31 | 0.11 | 0.088 |
| Cover vascular plants (scaled) | -0.13 | 138.09 | 0.07 | 0.065 |

**Figure S3. Output of LMM for effects of treatments on bryophyte species richness**


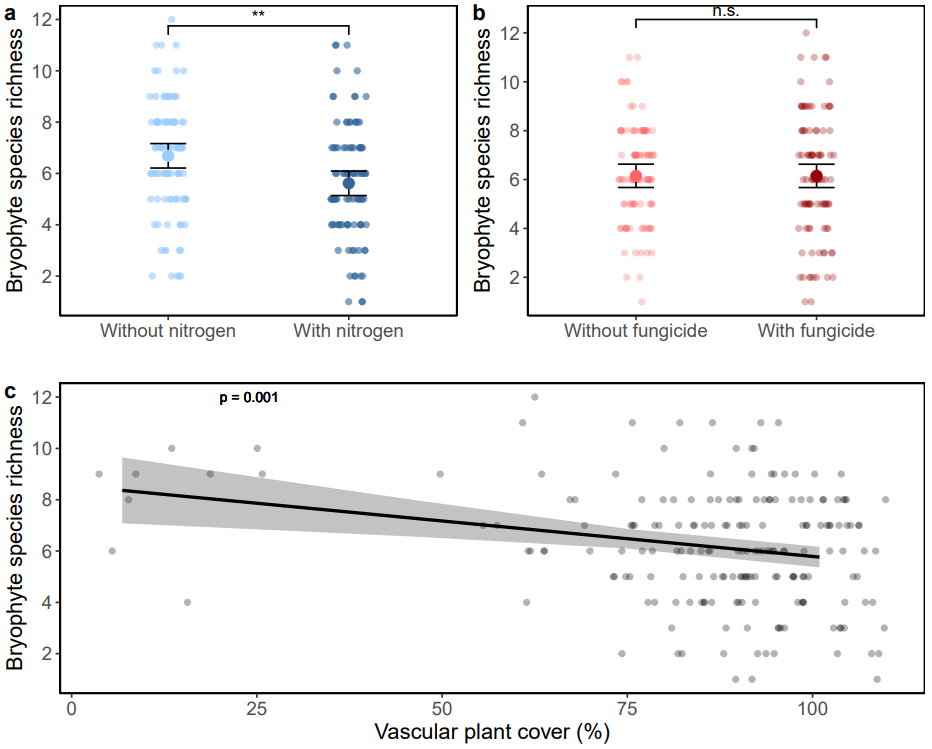
 **Figure S3**: Effects of a) nitrogen fertilization, b) fungicide application, and c) vascular plant cover (%) on **bryophyte species richness** of a subplot (output of LMM, n = 192). The effects of fungicide application were not significant. The ribbon and error bars represent 95 % confidence intervals. Raw data displayed as points. Estimates and CI were generated with the effects package [21,22]. n.s: not significant; **: 0.001 < p < 0.01.

**Figure S4. Output of LMM for effects of treatments on bryophyte cover**


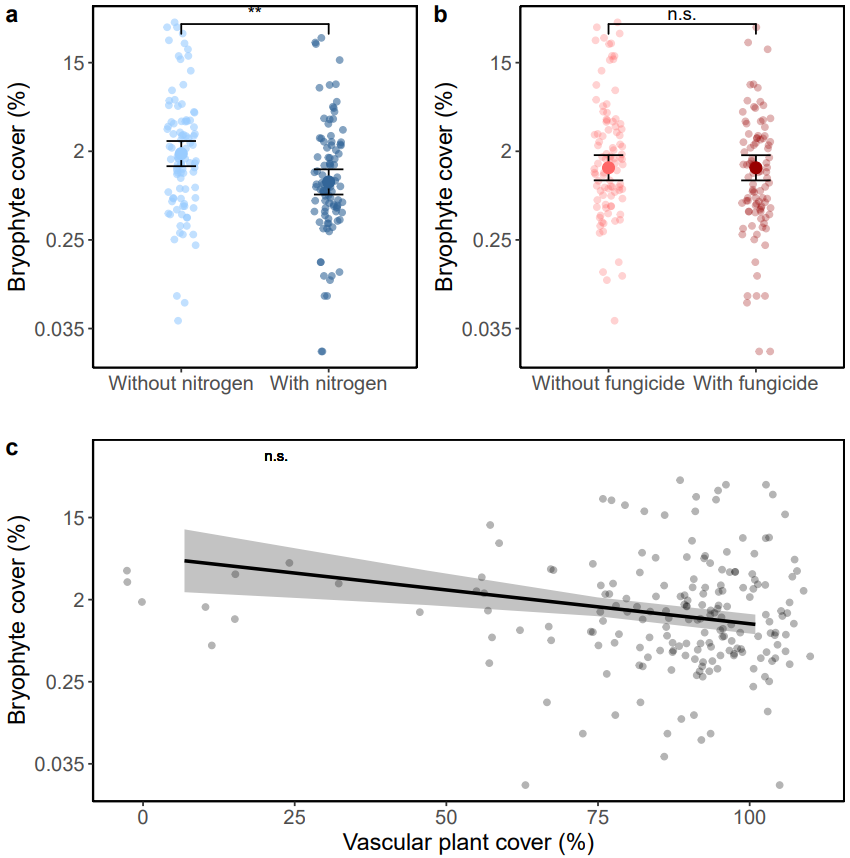


**Figure S4**: Effects of A) nitrogen fertilization, B) fungicide application, and C) vascular plant cover (%) on **bryophyte cover** of a subplot (output of LMM, n = 192). The ribbon and error bars represent 95 % confidence intervals. Raw data displayed as points. Estimates and CI were generated with the effects package [21,22]. n.s: not significant; **: 0.001 < p < 0.01.


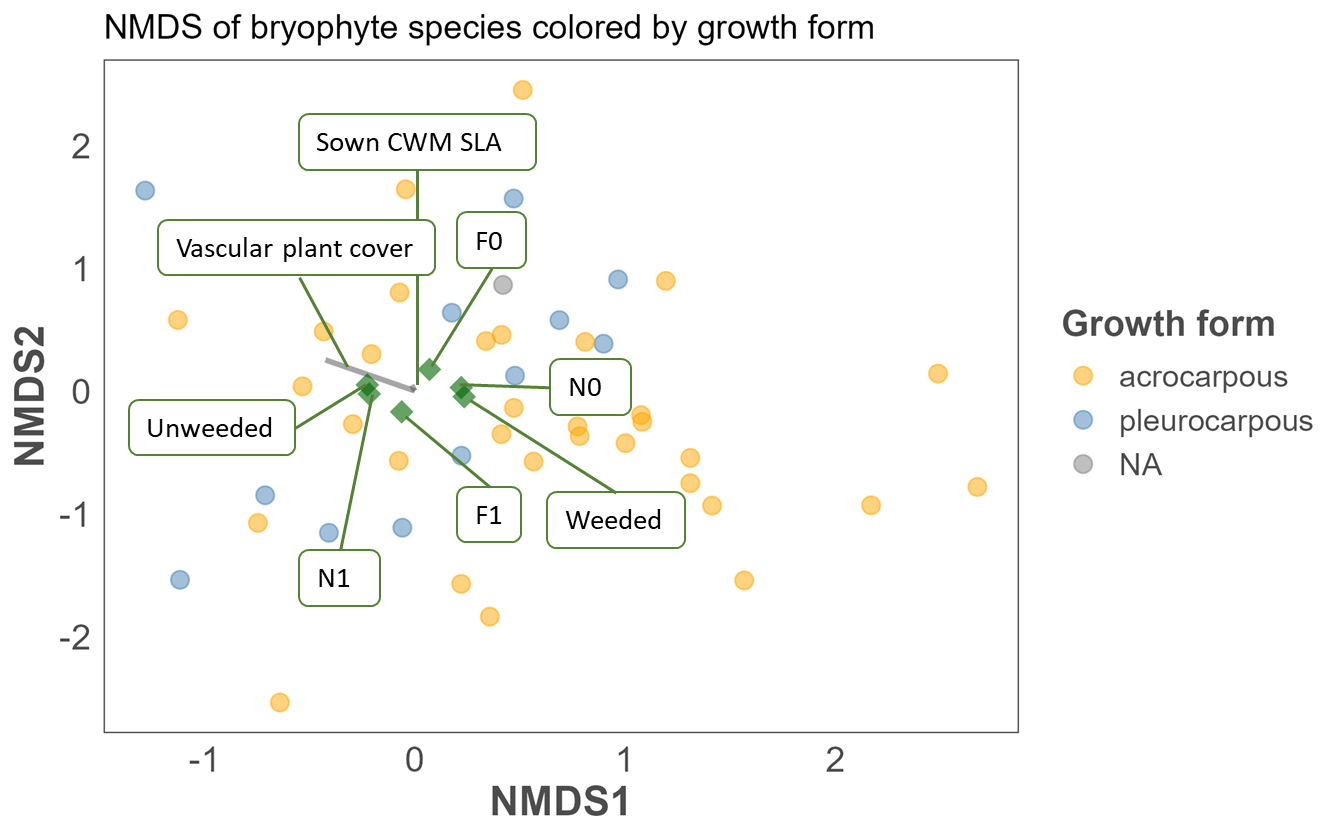


**Figure S5**: NMDS of the collected bryophyte species colored by growth form (acrocarpous, pleurocarpous, NA: liverwort species). Points represent the average of one species. Diamonds represent the effect of the categorical treatments (F0: no fungicide, F1: fungicide, N0: no nitrogen fertilization, N1: nitrogen fertilization, weeded, and unweeded) and grey lines the continuous ones (cover and sown community-weighted mean SLA of vascular plants).

**Table S7.** Summary of the linear mixed-effect models on the effect of nitrogen fertilization, fungicide application, weeding, and cover of vascular plants (scaled) on **a) cover of acrocarpous species, b) cover of pleurocarpous species, c) species richness of acrocarpous species, and d) species richness of pleurocarpous species**. Block, plot number, and the composition of vascular plants of each plot were fitted as random factors. Nitrogen, fungicide, and weeding were coded as factors. Bold font indicates statistical significance (at α = 0.05).

| **a) cover of acrocarpous species**   \| Fixed effects \| Estimate \| DF \| SE \| p \| \| --- \| --- \| --- \| --- \| --- \| \| (Intercept) \| -0.41 \| 122.42 \| 0.15 \| **0.006** \| \| Nitrogen \| -0.26 \| 93.83 \| 0.16 \| 0.105 \| \| Fungicide \| -0.21 \| 94.02 \| 0.16 \| 0.183 \| \| Weeding \| 0.08 \| 104.19 \| 0.12 \| 0.485 \| \| Cover vascular plants (scaled) \| -0.35 \| 168.88 \| 0.07 \| **< 0.001** \| | **b) cover of pleurocarpous species**   \| Fixed effects \| Estimate \| DF \| SE \| p \| \| --- \| --- \| --- \| --- \| --- \| \| (Intercept) \| -0.44 \| 104.32 \| 0.35 \| 0.204 \| \| Nitrogen \| -0.82 \| 64.70 \| 0.38 \| **0.034** \| \| Fungicide \| -0.47 \| 65.03 \| 0.38 \| 0.221 \| \| Weeding \| 0.77 \| 81.41 \| 0.29 \| **0.011** \| \| Cover vascular plants (scaled) \| 0.43 \| 122.97 \| 0.19 \| **0.026** \| |
| --- | --- | --- | --- | --- | --- | --- | --- | --- | --- | --- | --- | --- | --- | --- | --- | --- | --- | --- | --- | --- | --- | --- | --- | --- | --- | --- | --- | --- | --- | --- | --- | --- | --- | --- | --- | --- | --- | --- | --- | --- | --- | --- | --- | --- | --- | --- | --- | --- | --- | --- | --- | --- | --- | --- | --- | --- | --- | --- | --- | --- | --- |
| **c) species richness of acrocarpous species**   \| Fixed effects \| Estimate \| DF \| SE \| p \| \| --- \| --- \| --- \| --- \| --- \| \| (Intercept) \| 5.01 \| 129.50 \| 0.30 \| **< 0.001** \| \| Nitrogen \| -0.87 \| 91.04 \| 0.32 \| **0.008** \| \| Fungicide \| 0.02 \| 91.36 \| 0.32 \| 0.956 \| \| Weeding \| 0.89 \| 103.27 \| 0.27 \| **0.002** \| \| Cover vascular plants (scaled) \| -0.56 \| 180.67 \| 0.15 \| **< 0.001** \| | **d) species richness of pleurocarpous species**   \| Fixed effects \| Estimate \| DF \| SE \| p \| \| --- \| --- \| --- \| --- \| --- \| \| (Intercept) \| 1.09 \| 95.03 \| 0.12 \| **< 0.001** \| \| Nitrogen \| -0.23 \| 64.48 \| 0.12 \| 0.053 \| \| Fungicide \| -0.04 \| 64.96 \| 0.12 \| 0.715 \| \| Weeding \| 0.31 \| 98.04 \| 0.09 \| **0.001** \| \| Cover vascular plants (scaled) \| 0.04 \| 153.89 \| 0.06 \| 0.429 \| |


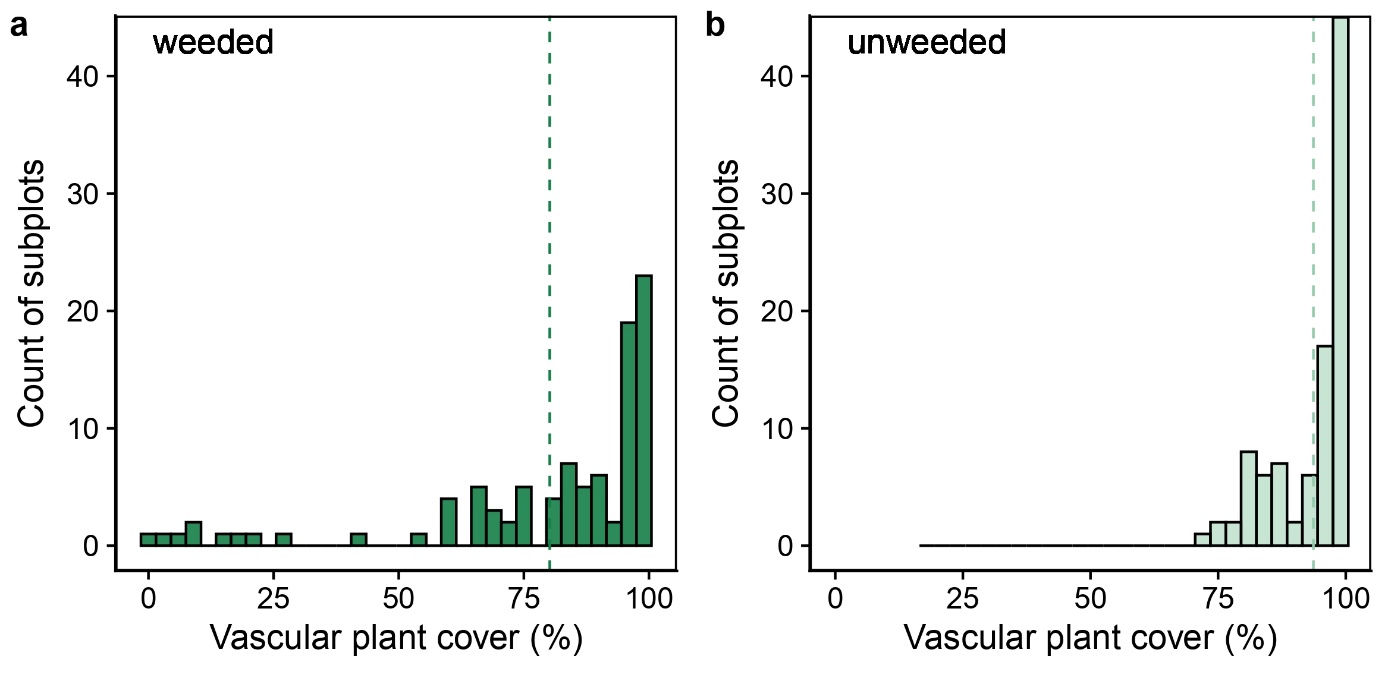


**Figure S6**: Percentage cover of vascular plants in the a) weeded and b) unweeded subplots. The dotted lines are the mean value for each group (weeded = 80 %, unweeded = 94 %).


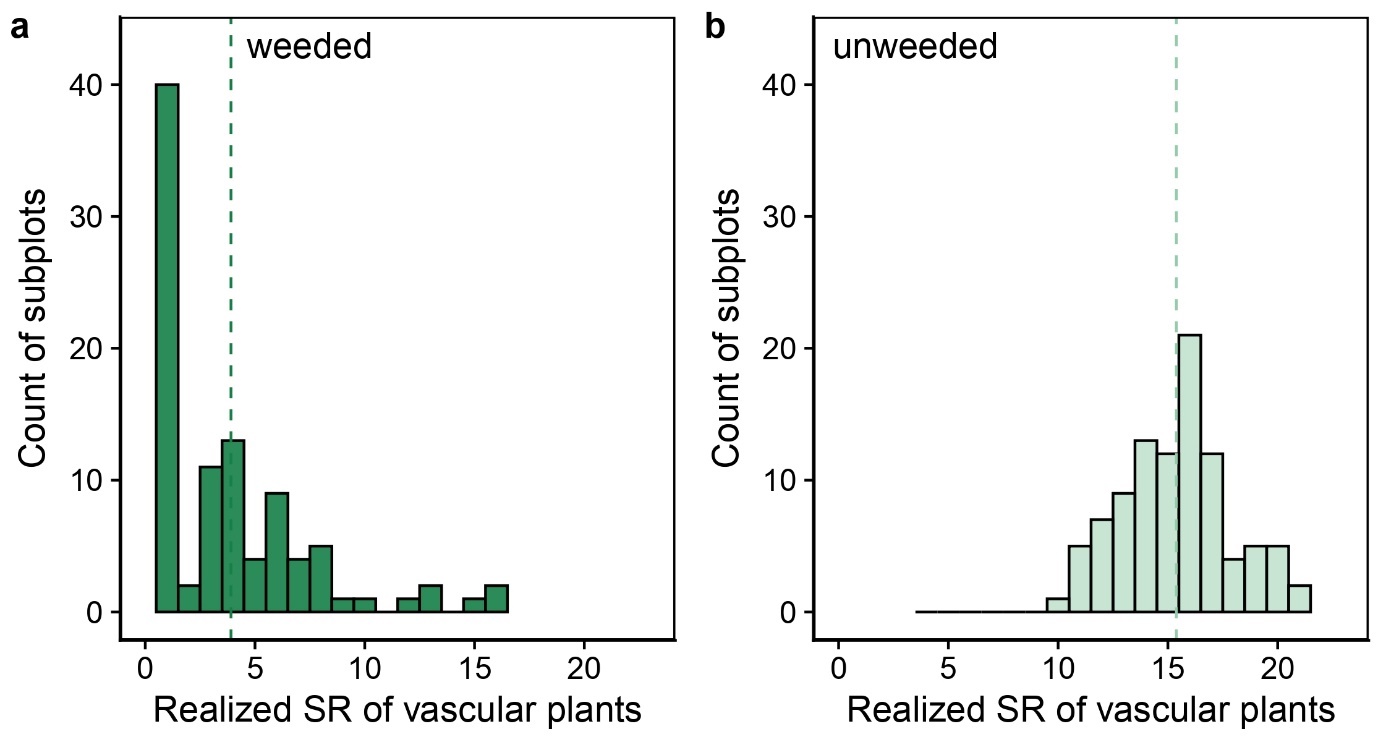


**Figure S7**: Realized species richness (SR) of vascular plants in the a) weeded and b) unweeded subplots. The dotted lines are the mean value for each group (weeded = 4, unweeded = 15).

# Supplementary References

1. R Core Team. R: A Language and Environment for Statistical Computing. Preprint at https://www.R-project.org/ (2023).

2. Hijmans, R. J. terra: Spatial Data Analysis. Preprint at https://CRAN.R-project.org/package=terra (2023).

3. Juillerat, P. *et al.* Flora Helvetica Checklist. (2017) 10.5167/uzh-165877.

4. Oesau, A. & Augustin, B. Nebenwirkungen von Herbiziden und Fungiziden auf die Artenvielfalt der Moosflora in Obst- und Rebanlagen. *Journal of Plant Diseases and Protection* **Sonderheft 19**, 935–942 (2004).

5. Cappelli, S. L., Pichon, N. A., Kempel, A. & Allan, E. Sick plants in grassland communities: a growth-defense trade-off is the main driver of fungal pathogen abundance. *Ecol Lett* **23**, 1349–1359 (2020).

6. Pichon, N. A., Cappelli, S. L. & Allan, E. Intraspecific trait changes have large impacts on community functional composition but do not affect ecosystem function. *Journal of Ecology* **110**, 644–658 (2022).

7. Helbach, J., Frey, J., Messier, C., Mörsdorf, M. & Scherer-Lorenzen, M. Light heterogeneity affects understory plant species richness in temperate forests supporting the heterogeneity–diversity hypothesis. *Ecol Evol* **12**, (2022).

8. Walde, M. *et al.* Both diversity and functional composition affect productivity and water use efficiency in experimental temperate grasslands. *Journal of Ecology* **109**, 3877–3891 (2021).

9. Cappelli, S. L., Pichon, N. A., Mannall, T. & Allan, E. Partitioning the effects of plant diversity on ecosystem functions at different trophic levels. *Ecol Monogr* **92**, (2022).

10. Arróniz-Crespo, M., Leake, J. R., Horton, P. & Phoenix, G. K. Bryophyte physiological responses to, and recovery from, long-term nitrogen deposition and phosphorus fertilisation in acidic grassland. *New Phytologist* **180**, 864–874 (2008).

11. Boch, S. *et al.* Direct and indirect effects of land use on bryophytes in grasslands. *Science of the Total Environment* **644**, 60–67 (2018).

12. Bergamini, A. & Pauli, D. Effects of increased nutrient supply on bryophytes in montane calcareous fens. *J Bryol* **23**, 331–339 (2001).

13. Pichon, N. A. *et al.* Nitrogen availability and plant functional composition modify biodiversity-multifunctionality relationships. *Ecol Lett* **27**, (2024).

14. Pichon, N. A. & Cappelli, S. L. Nitrogen availability and plant functional composition modify biodiversity-multifunctionality relationships. (2020) 10.1101/2020.08.17.254086.

15. Mitchell, C. E., Reich, P. B., Tilman, D. & Groth, J. V. Effects of elevated CO2, nitrogen deposition, and decreased species diversity on foliar fungal plant disease. *Glob Chang Biol* **9**, 438–451 (2003).

16. Stevens, C. J., Dise, N. B., Mountford, J. O. & Gowing, D. J. Impact of Nitrogen Deposition on the Species Richness of Grasslands. *Science (1979)* **303**, 1876–1879 (2004).

17. Ochoa-Hueso, R. & Manrique, E. Effects of nitrogen deposition on growth and physiology of Pleurochaete squarrosa (Brid.) Lindb.; A terricolous moss from mediterranean ecosystems. *Water Air Soil Pollut* **224**, (2013).

18. Löbel, S., Dengler, J. & Hobohm, C. Species richness of vascular plants, bryophytes and lichens in dry grasslands: The effects of environment, landscape structure and competition. *Folia Geobot* **41**, 377–393 (2006).

19. Hodgetts, N. G. *et al.* An annotated checklist of bryophytes of Europe, Macaronesia and Cyprus. *J Bryol* **42**, 1–116 (2020).

20. Meusel, H. Wuchsformen und Wuchstypen der europaischen Laubmoose. *Nova Acta Leopoldina* **3**, 123–277 (1935).

21. Fox, J. & Weisberg, S. *An R Companion to Applied Regression*. (Sage publications, 2019).

22. Fox, J. & Weisberg, S. Visualizing fit and lack of fit in complex regression models with predictor effect plots and partial residuals. *J Stat Softw* **87**, 1–27 (2018).
